# Diseases of the Corpuscles of Blood

**Published:** 1859-12

**Authors:** 


### SELECTIONS.

### *Diseases of the Corpuscles of Blood.*

(From the *Medical News and Library.*)

With respect to disease or abnormal metamorphosis of the corpuscles of the blood, and its association with symptoms of fever, numerous observers have described their darker color, and the easy transudation of their coloring matter in typhus. Denis, for example, describes the blood in typhus fever as deficient in fibrin. He says that air had no effect in reddening it; and, on analysis, it was found to contain ammonia. Dr. Armstrong observed the blood in typhus from the temporal artery as dark as that from the veins.

The general appearance of blood in malignant fevers, has been described by Huxham and Fordyce. At the first commencement of symptoms of fever, the blood was sometimes buffed; but the clot beneath was always of a loose texture, scarcely cohering, and very dark in color. If the patient was bled two or three days after the onset of fever, the blood was found incoagulable, having the appearance as when spirits of hartshorn is added to blood as it runs from the vein, which darkens its color, and prevents coagulation.

In the yellow fever of the West Indies, blood has been observed to be hotter than in health. As fever progresses, it becomes black and thin. Dr. Blair says that, in many instances, the corpuscles were found so much dissolved, that, in several specimens of fever-blood, but few of them could be observed.

It has been found that a diminished amount of carbonic acid is discharged from the blood by respiration in cases of fever; and this fact, taken in conjunction with the dark color of blood, is conclusive as to one, at least, of the functions of the corpuscles being disordered in fever.

Rokitansky, Simon, Perry, and many other accurate observers, all speak of the dark color and changed state of the corpuscles of blood in malignant fever, of the incoagulability of the plasma, and of the staining of the tissues and of the serum by the coloring matter which transudes the corpuscles.

As regards the microscopical appearances of the corpuscles of blood in fever, we hesitate at present to lay any stress upon them; because in persons in health, they very speedily change their figure and outline in a very uncertain manner. Some of them became notched with projecting points, and otherwise changed in outline; others assume globular forms; they are influenced as respects these changes, it would appear, by the temperature of the glass upon which they are received for examination, and by the way in which they are covered by the thin glass. Some are seen paler, and some smaller than others; some adhere closely together in rolls, others float about separately without the least disposition to adhere to their neighbors. All these varieties may be seen in the same small quantity of blood which is alone available for microscopic inspection, especially if the exterior edges of the film of blood be brought into the focus of the microscope.

Notwithstanding these obstacles to the drawing any positive conclusion from the microscopical appearances of the blood-corpuscles in cases of fever, it is quite as likely to be by the microscope as by chemistry that future advances in the pathology of these bodies will be made. For how can any bulky chemical manipulation satisfactorily eliminate results from the plasma from results from the corpuscles? especially if, under the influence of reagents, as we shall show, they throw out matter from their interior into the fluid in which they swim, and yet preserve their integrity or individuality. "Hitherto, at all events," as Rokitansky has well observed, "chemistry cannot be said to have excelled, as respects the pathology of these bodies, the achievements of a circumspect anatomical survey, notwithstanding the limited resources at the disposal of the latter."

*Experiment* 1.—Take a slip of glass, such as is used for mounting microscopical objects, receive on it a very small drop of blood, and place close to it with a pipette a drop of any fluid, chloroform, ale, weak sugar and water, etc., and the quantity of the extraneous fluid should not exceed the quantity of blood. Upon now dropping on the two fluids a thin piece of glass, their nearest edges will mingle, but in various proportions.

In these experiments we find the *outline* and the *interior* of the corpuscles of extremely various appearance in the same experiment, but nothing appears thrown out from them.

*Experiment* 2.—Proceed as in the last experiment, but let the fluid used be sherry wine. The corpuscles, along the line where the blood and wine mingle, will soon begin to throw out molecules around their circumference, many of which pass away into the fluid; others grow out into long tails, which remain attached to the corpuscles, and terminate in a knob. They also wave about in a very singular manner. After some time (half an hour), the tails or filaments become nodulated, and then break away from the corpuscles, and when they have done so, they continue their singular movements in the fluid.

Some years ago, when making observations on the plasma or liquor sanguinis of blood, drawn by venesection in cases of fever and inflammation, we observed in the fluid a vast multitude of minute molecules. (*Medical Gazette*, vol. ii., 1841–42.) And the molecules seen in the experiment here related, are precisely the same in magnitude and appearance as those we saw in the fluid of blood drawn in the cases of fever. Now we believe that the blood-corpuscles do not immediately lose their vital or chemical properties when withdrawn from the body. Therefore, we regard the remarkable forms and actions they exhibit under the influence of sherry wine, as phenomena of a species of reaction, which, amongst multitudes of them, is various or unequal, hence the various appearances presented. In some, the resistance offered to the extraneous fluid is more easily overcome than in others.

We have frequently examined with the microscope blood taken from persons affected with scarlet fever, with reference to the appearances in the plasma, and have always noticed the following facts. The colorless or plasma corpuscles are much more numerous than in persons in health, and especially so if the blood be drawn from the skin as the fever is passing off, and the epidermis beginning to exfoliate. They are of different sizes and present different appearances. In the open spaces between the rolls of red corpuscles, irregular masses of granular matter and numerous free molecules are seen floating in the fluid.

In cases of diphtheria, we find that the plasma presents the same appearances as are seen in scarlet fever. Formerly we supposed the free molecules observed in the plasma of blood, drawn in cases of fever and inflammation, to be derived from the colorless corpuscles; but now that we have seen them thrown out by the red corpuscles, there is actual proof that these corpuscles, in their reactions upon extraneous substances, do themselves throw off matter into the fluid in which they swim. The appearances, then, which we have seen in the fluid of the blood in cases of fever, and the behaviour of the corpuscles in the experiment we have related, appear to corroborate our conclusions respecting the excretions of the corpuscles passing into the plasma, and the association of these excretions with phenomena of fever.

Before proceeding with our argument, it will be convenient now to refer to some collateral incidents which require our notice.

First, it may be objected to our proposition respecting the antecedent of fever, namely, disorder or disease of the blood-corpuscles, that in venosity of blood, where a poisonous element of the corpuscles fails of being discharged by respiration, the symptoms are those of brain disturbance, and not of fever. In *morbus cœaruleus* there is no fever necessarily.

To this objection it may be replied, that the presence of a venous quality in the corpuscles does not imply disorder or disease in them, in the same way that it is concluded to arise from miasms in the air. The substance—carbon or other matter—which gives to the corpuscles their venous character, is a substance natural to them, an essential ingredient of their composition in certain parts of their course in the circulation. Therefore, it is to be expected, it would occasion no unusual reaction on the part of the corpuscles themselves, though the brain suffers; whereas, a poison from the air may be presumed something quite heterologous to the corpuscles, and doubtless they react with more energy against a foreign injurious matter, than against anything which is a part of their normal composition.

In cases of blood-poisoning through the stomach, such as drunkenness from alcohol, narcotism from opium, and salivation from mercury, the locality and character of the symptoms point out which organ it is that suffers first or most from a particular poison diffused through the plasma. And if in these examples fever be absent, the argument is, that the parenchymatous organ is affected before the corpuscles of the blood, fever appearing when they partake of the disorder.

In cases of blood-poisoning through the lungs, on the contrary, the symptoms begin with fever, and the argument is, that the corpuscles of blood are here affected first, before the plasma or any of the local parenchymata; fever denoting an abnormal metamorphosis of the corpuscles.

A venous state of the corpuscles of blood is not an abnormal metamorphosis. The condition comprehended in the term venous is one natural to them. Misplaced venous corpuscles disorder the brain; but fever does not appear, because the venous state is natural to the corpuscles. The absence of symptoms of fever, when venous blood circulates arterially, is therefore no valid objection to the proposition we are arguing.

Again, we have hitherto purposely abstained from any reference to the brain or nervous system as causes of fever and inflammation, not because we underrate their powerful influence over blood and the secretions, but because so important a part of our subject requires a special consideration, which we can here but briefly indicate.

It has been argued in the former lecture, that the elements of the parenchymatous organs and the corpuscles of blood have the common properties of other living cellular bodies; and among these are properties of affinity, which differ in relation to different substances. And there can be no doubt that the corpuscles of the blood, as respects the various substances they encounter in their course during circulation have much more intimate relations with (or a greater affinity for) some than others. For example, in the lung, a special reciprocal action takes place between the corpuscles which are within the vessels and the air which is outside them. And, as if to facilitate this action, the coats of the bloodvessels in the cells of the lung are reduced to an extreme degree of thinness.

Now, it may be argued of any other special organ, where the coats of the capillary vessels are reduced to a still greater degree of thinness than in the lung, that they are so for a similar purpose. Thus, in the brain, an organ very largely supplied with blood, the coats of the extreme vessels are so thin that we fail to trace them in all their various ramifications; indeed, so entirely are they altered that the elements of the organ have but a slight coherency. And, unless the contrary can be shown, we may infer this disappearance of the ordinary properties of bloodvessels to be for the purpose of removing all obstacle to the closest possible contact between the corpuscles of the blood and the parenchyma of the brain. This inference is corroborated by the fact that the brain is the organ specially affected by an abnormal circulation of venous corpuscles. That is to say, the brain is the organ which first detects the presence of an abnormal venous quality in the corpuscles.

If, then, we may regard the thinning away of the coats of the capillaries of the brain as facilitating a contact action between the corpuscles of blood and the substance of the brain; and if, moreover, we are able to appeal to the known effect of venous corpuscles upon the functions of the brain denoted by drowsiness, stupor, delirium, and coma, as proof of a special action between the brain and the corpuscles, then we may claim the constant occurrence of similar cerebral disturbances in fever as corroborative of the view which bases phenomena of fever upon abnormal metamorphosis of the corpuscles of blood; the brain—to use a chemical phrase—being the test of the condition of the corpuscles.

All the functions of a living body may be comprehended as a series of actions and reactions; and, if it be admitted that the corpuscles of blood do perform a special function in the brain, there must necessarily be reaction from the brain upon the corpuscles.

CASE.—A young married woman, aged 19, was delivered of her first child. The labor was natural, and she went on favorably until the fourth day from her confinement, when her husband stayed out late at night, and returned home drunk from a fair, very much knocked about. By this she was thrown into a state of great nervous excitement. Very shortly after, she was seized with a numbness of the legs and shivering, and then with pain in the head and wandering of the mind. The secretion of milk was interrupted; the skin became hot and dry; no sleep could be procured; and the pulse beat 120 in the minute. The wandering of the mind passed into furious delirium, and all the symptoms of fever and excitement continued for several days. It was only by judicious medical treatment and careful nursing that the disorder was subdued on the seventeenth day.

In this case, we argue that the fever arose, not immediately from the nervous shock, but from disorder of the blood-corpuscles; and, as our researches have led us to interpose distemperatures of the fluid of the blood between errors in diet and inflammation, and disorder of the corpuscles of blood between aerial miasms and fever, so analogously of mental emotions and nervous shocks, when they occasion or aggravate fever or inflammation, we conclude they do so by disordering, or adding to the disorder, of one or other, or both parts of the blood. Errors in diet do not produce inflammation, unless the fluid of the blood be distempered; also a miasmatous air does not produce fever, except the blood-corpuscles be affected; so, too, great convulsions and loss of consciousness (in epilepsy, etc.) pass away without fever, if the blood be not affected. The close sympathy between states of the corpuscles of blood and the brain, then, supports our proposition that symptoms of fever and the generation of poisons in the blood are to be based upon disorder of the corpuscles.

